# The long-term impact of wheelchair delivery on the lives of people with disabilities in three countries of the world

**DOI:** 10.4102/ajod.v6i0.344

**Published:** 2017-09-08

**Authors:** Susan Shore

**Affiliations:** 1 Department of Physical Therapy, Azusa Pacific University, United States

## Abstract

**Background:**

Lack of access to mobility for people with disabilities, particularly in less- resourced settings, continues to be widespread. Despite challenges to wheelchair delivery, the benefits to health, employment, social integration and life satisfaction are apparent.

**Objectives:**

Previous studies have explored the impact of receiving a wheelchair on the lives of the users through cross-sectional or short-term longitudinal analysis. The current study was undertaken to evaluate whether previously reported changes were sustained after 30 months of use, and whether results varied between two differing models of a wheelchair.

**Method:**

One hundred and ninety-one subjects from Peru, Uganda and Vietnam received one of two models of wheelchair provided by the Free Wheelchair Mission. Using interviews to record survey results, data were collected at the time the wheelchair was received and following 12 and 30 months of use. Variables of overall health, employment, income and travel were explored through non-parametric analysis.

**Results:**

There was a significant improvement in overall health and distance travelled after 12 months, but these changes were no longer significant by 30 months (Friedman test for overall change, *p* = 0.000). Employment status showed a small but significant increase at 12 and 30 months (Cochran’s Q, *p* = 0.000). Reported income increased slowly, becoming significantly different at 30 months (Friedman test, *p* = 0.033). There was no association between the model of wheelchair received and the incidence of pressure ulcers, pain or maintenance required. There was higher satisfaction with the GEN_2 wheelchair at 12 months (*p* = 0.004), but this difference was not apparent by 30 months. Overall wheelchair satisfaction and maintenance levels were favourable.

**Conclusion:**

While overall health status, and distance travelled into the community fluctuated over time, receipt of one of two models of a wheelchair in less-resourced settings of the world appears to have a positive sustained impact on employment and income. Further investigations should be carried out to confirm these results and explore the factors responsible for fluctuating variables. This study affirms the importance of long-term follow-up of outcomes associated with wheelchair distribution in less-resourced environments.

## Introduction

With the signing of the United Nations Convention on the Rights of Persons with Disabilities (UNCRPD) in 2006, mobility, and the provision of assistive technology for those who lack it, was recognised as a human right (Borg, Larsson & Östergren 2011). Despite the efforts of both national and international government and non-governmental organisations to provide access to mobility for the world’s people with disabilities, approximately 85%–95% of those who need a wheelchair do not have one (WHO 2008). This is accompanied by diminished access to education, employment and medical care (WHO 2011).

The challenges related to wheelchair provision in less-resourced countries include limited state services to systematically identify those in need, a lack of medical and rehabilitation services to accompany assistive technology (Tasiemski, Priebe & Wilski 2013), and a shortage of personal finances to afford it (Mitra, Posarac & Vick 2012). The shortage of wheelchairs is compounded by the lack of accessibility in construction and transportation services. Physical barriers for wheelchair use include narrow doorways, steep ramps and inaccessible bathrooms (Pearlman et al. 2006). There is an additional requirement to be able withstand extreme weather and poor road conditions. This is exemplified by the report of Tasiemski et al. (2013) that travel time from the residence of subjects with spinal cord injury to the nearest rehabilitation centre averaged 4.29 h in India, 2.77 h in Vietnam and 3.02 h in Sri Lanka, thus limiting the opportunity for service acquisition.

Access to a wheelchair has been shown to impact both the social and health needs of the user. In a review of the provision of services for children with disabilities, both children and parents placed the highest priority on independence, and the psychosocial outcomes associated with wheelchair interventions. This included the development of social skills and the ability to participate in broader society (Bray et al. 2014). Rousseau-Harrison and Rochette (2013) found that acquiring a wheelchair tended to improve a child’s participation in social relationships, self-care, play, and mobility.

Women in Indonesia, who received a wheelchair in conjunction with the WHO eight-step approach to wheelchair distribution, reported a better quality of life than those on a waiting list (Toro et al. 2016). Although there was no difference in the number of pressure sores or employment status between those who received a chair and those on the waiting list, both men and women reported improved environmental health scores (health and financial resources).

People with disabilities are at increased risk for lower income and higher poverty levels (Mitra et al. 2012; Mizunoya & Mitra 2013), made worse by a decreased ability to convert what is available into an adequate standard of living (Shore & Juillerat 2012). A cyclic relationship exists between disability and poverty, accompanied by social and cultural exclusion and denial of opportunities for economic development (Harkins, McGarry & Buis 2012). Conversely, patients who are employed after spinal cord injury have increased life satisfaction (Schonherr et al. 2005; Van Koppenhagen et al. 2008) associated with increased opportunity for socialisation, productivity and income.

While the causes of poverty in this population are multi-faceted (Eide & Ingstad 2013), increased mobility through provision of a wheelchair appears to have a positive impact. Twelve months after receipt of a wheelchair, 519 users in India, Chile and Peru reported a small but statistically significant increase in health, mood state, independence and employment (Shore & Juillerat 2012). Wheelchair provision in Ethiopia was accompanied by a shift in time expenditure towards economic productivity, with less time spent on begging and more time spent on other income-generating activity (Grider & Wydick 2015).

The challenges and benefits of wheelchair provision continue to be studied even as more models of both chairs and service provision emerge. As demonstrated by Visagie et al. (2015), results vary depending on function, durability and fit of the wheelchair, making it imperative to continue to monitor outcomes.

While previous studies have examined the variables of employment, health and life satisfaction following receipt of a wheelchair in less-resourced countries, they have used either a cross-sectional or a short-term (one year) longitudinal approach. The current study was undertaken to assess whether reported changes in previous studies were both verified and sustained over a 2.5 year period, and whether the model of wheelchair played a role in the results.

## Research methods

### Design

The current study was designed by the Free Wheelchair Mission (FWM) organisation, modifying surveys used in previously published studies (Shore 2008; Shore & Juillerat 2012). The survey questions used are listed in Appendices 1 and 2. Approval for the study was obtained through the organisation’s internal review process using methodology similar to that previously reported, with local partners ensuring that cultural norms, ethics and policies were adhered to. Following collection of data, it was released to the author for analysis.

Two hundred prospective wheelchair recipients in each of three countries (Peru, Uganda and Vietnam) were identified by local affiliates of the FWM using community social workers and databases. Subjects were given the option to participate in the study, but received a wheelchair at no cost, regardless of participation.

Because 57.2% of all participants reported a maximum of 3rd grade or lower education (including 37.8% with no formal education), willingness to participate was considered consent. All volunteers were accepted without exclusion, and parents were allowed to complete the survey on behalf of their children.

Independent contractors were hired and trained in survey procedures by employees of the FWM. The contractors were nationals, who had previously worked for national and international firms, and were fluent in local languages and customs. They were responsible for translation of the surveys from English into Spanish/Vietnamese/Swahili. Subjects were interviewed before they received the wheelchair, and at 12 and 30 months afterwards.

### The wheelchairs

Subjects received one of two models of wheelchairs based on availability and choice of the local affiliate, rather than user characteristics. The wheelchairs are products of the FWM, designed for 3–5 years of use in rugged terrain when appropriately maintained (FWM 2016). They are manufactured in China and shipped to local partner organisations around the world where they are assembled and distributed.

Each wheelchair is distributed with a flat covered 50 mm polyurethane foam cushion, a wrench, air pump and a patch kit for tyres. Included with each chair is a written manual (in Spanish, Vietnamese and English) describing proper use and care, including safe transfers, exercises for strength and range of motion, and the prevention of pressure ulcers. It includes contact information for the partner organisation, should replacement parts be required. For the GEN_2 wheelchair, local affiliates were expected to measure the user, choose the size of wheelchair and adjust it for fit. For both GEN_1 and GEN_2 users, they provide basic training to the wheelchair user or family members at the time the chair is given. Wheelchairs are provided free of charge to recipients, made possible through local and national fundraising efforts.

**FIGURE 1:**
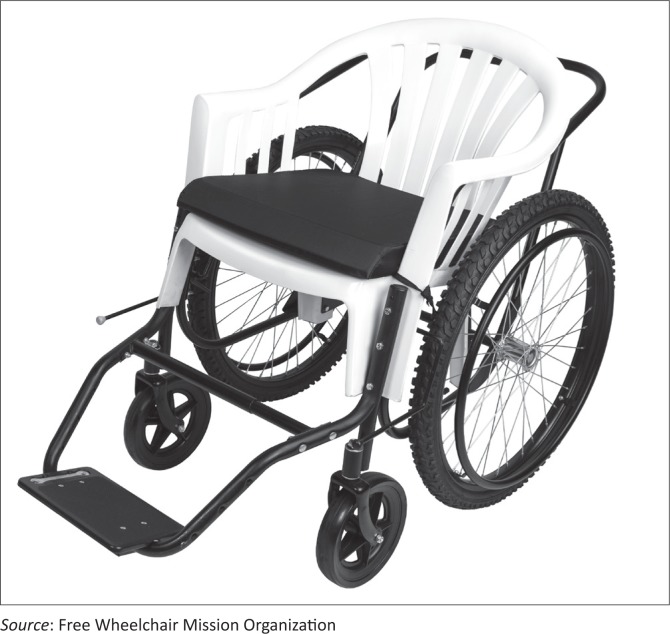
The FWM GEN_1 wheelchair.

The GEN_1 wheelchair (Figure 1) is a one size (Table 1), non-adjustable chair constructed with a polypropylene seat and back on a rigid frame. It has 8-inch natural rubber castors in the front and 24-inch medium tread pneumatic tyres in the back. It includes a five-point adjustable over-the-shoulder harness for postural support as needed, independent steel-locking rear wheel brakes with extended length handles, and one large adjustable footrest. It weighs 17.6 kg (38.8 pounds).

**TABLE 1:** Dimensions of wheelchair models and sizes.

Model of wheelchair	Seat width (cm)	Seat depth (cm)	Back height (cm)
GEN_1	44 (front), 41 (rear)	39.5	43
GEN_2 Small	34	27–44	34–46
GEN_2 Medium	42	27–44	34–46
GEN_2 Large	49	27–44	34–46

*Source*: Author’s own work

**FIGURE 2:**
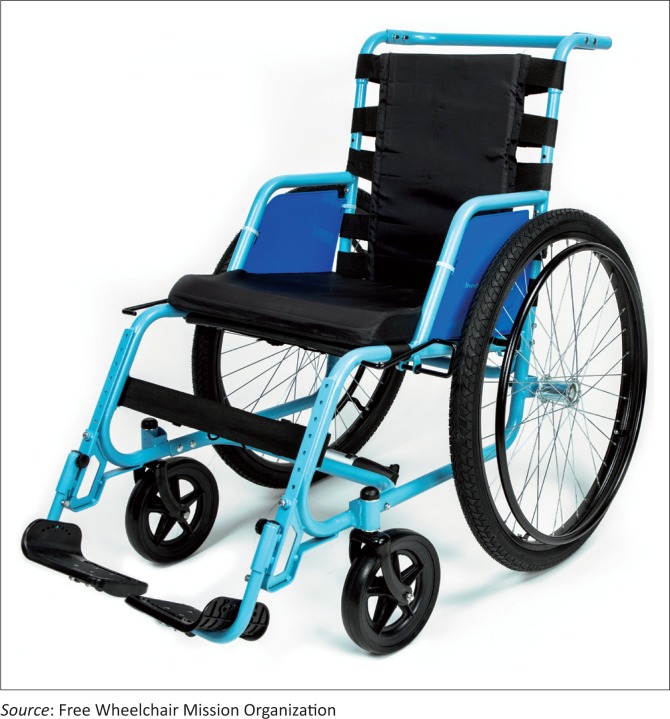
The FWM GEN_2 wheelchair.

The GEN_2 wheelchair (Figure 2) is fully adjustable, approved by the United States Federal Drug Administration, and comes in three sizes (Table 1). It has two swing-away footrests, a rigid frame and a rigid moulded seat. Leg rests can be individually adjusted to accommodate differing leg lengths and angled as needed. Seat depth and back height are customisable to fit the user in all chairs. Rear tyres are 26-inch medium tread pneumatics with 3 degree camber; front tyres are large rubber castors. Brakes are independent, steel-locking with extended length handles. Net weight of the chair is 16.5 kg (36.4 pounds). The seat is attached to a steel frame by polypropylene webbing straps secured with parachute cord. The backrest is comprised of EVA foam padding inside a water-resistant, fire-retardant nylon cover.

### Subjects

Wheelchair recipients from three different countries and continents participated in the study: Peru, Uganda and Vietnam. The study began with 596 subjects, with approximately 33% from each of three countries. No data were kept on those who chose not to participate. Demographics of the original sample by country are included in Table 2. At the end of 30 months (the final survey), subjects were excluded who did not complete all three surveys or who reported using more than one wheelchair. Following attrition and exclusion, the demographics of the study sample (*n* = 191) are seen in Table 3.

**TABLE 2:** Demographics of original sample by country.

Characteristics	Peru, *n* = 215	Vietnam, *n* = 188	Uganda, *n* = 193
Overall health rating			
Mean ± (SD)	6.13 ± (1.36)	4.30 ± (1.10)	6.20 ± (2.18)
Median + (IQ range)	6.00 + (2.00)	4.00 + (1.75)	6.00 + (4.00)
Age (years)			
Mean + (SD)	58.37 + (16.43)	65.14 + (17.38)	38.69 +(26.71)
Percentage (number) of sample reporting adequate amount and nutrients in the daily diet	89.8% (193)	26.1% (49)	69.4% (134)
Percentage (number) of sample that never went outside the home	3.3% (7)	69.7% (131)	34.2% (66)
Percentage (number) of sample reporting skin ulcers in the last 12 months	15.3% (33)	8.0% (15)	9.0% (17)

*Source*: Author’s own work

**TABLE 3:** Demographics of study sample.

Characteristics	Frequency / Descriptive *n* = 191
Percentage (number) of total samples by country	
Peru	51.3% (98.00)
Uganda	35.1% (67.00)
Vietnam	13.6% (26.00)
Home setting	
Large metropolitan area	44.5% (85.00)
Rural	17.3% (33.00)
Village or small town	38.2% (73.00)
Gender	
Men	41.4% (79.00)
Women	58.6% (112.00)
Age	
Mean ± (SD)	38.81 ± (27.31)
Range	3.0–98.0
Percentage (number) diagnosis	
Stroke	6.3% (12.00)
Cerebral palsy	16.8% (32.00)
Spinal cord injury	2.6% (6.00)
Polio	13.1% (25.00)
Trauma/fracture	11.1% (21.00)
Amputee	5.2% (10.00)
Arthritis	5.8% (11.00)
Other childhood (e.g. club foot, hydrocephalus)	8.9% (17.00)
Other general (e.g. Parkinson’s, muscular dystrophy)	30.2% (58.00)
Model of wheelchair received by country and overall	
Three countries combined *n* = 188	GEN_1 51.6% (97.00)
	GEN_2 48.4% (91.00)
Peru *n* = 98	GEN_1 74.5% (73.00)
	GEN_2 25.5% (25.00)
Uganda *n* = 64	GEN_1 29.7% (19.00)
	GEN_2 70.3% (45.00)
Vietnam *n* = 26	GEN_1 19.2% (5.00)
	GEN_2 8.8% (21.00)

*Source*: Author’s own work

### Data analysis

Following the interviews, survey data were entered electronically by local contractors into Survey Monkey®. Results were exported into SPSS PASW 23.0 in the United States. All detailed names and addresses were kept in separate files by the FWM. Analysis was completed using subject numbers only. Data from all three countries were combined into one file for analysis, with subject numbers being followed across three surveys. Results were compared to individual country data and can be assumed to conform to final patterns unless otherwise reported.

Descriptive data (means, medians, standard deviations, interquartile range and frequencies) were obtained from 191 subjects at baseline, 12 and 30 months. Dichotomous variables (employment, presence of pain, presence of pressure ulcers in the previous 12 months) were analysed across the three time points using Cochran’s Q with McNemar’s test for *post hoc* review at a significance level of 0.05.

The Friedman test was used to assess differences across surveys for ordinal data (overall health, distance travelled per day and family income) with Wilcoxon Signed Rank used for *post hoc* analysis.

Wilcoxon Signed Rank was used to assess differences in rank data collected at only two time periods (satisfaction with the wheelchair at 12 and 30 months, and independence level at baseline and 30 months). Pearson chi square test for independence was performed using crosstabs to assess the association between the model of wheelchair received with the presence of pressure ulcers, employment, pain, mechanical breakdown, and satisfaction at 12 and 30 months. The significance level for all tests was set at 0.05.

## Results

### Subjects

From the original sample (596 subjects), the death rate was 18.37%, greatest in Vietnam. At 30 months, 5.56% of participants could not be located, and 11.87% of wheelchairs had been sold, stolen or given away. On the 30-month survey, those subjects who reported having received or purchased an additional wheelchair from other sources during the course of the study were excluded from analysis to avoid confounding results. Subjects who did not complete surveys at all three time periods were also excluded. Together, these exclusions accounted for 32.15% of the original data. Therefore, 191 subjects (32.05% of the original sample) made up the study sample; subjects in Peru comprised over 50% of the study sample.

### Health status

While the overall health ranking (0–10) changed significantly over time (Freidman test, *p* = 0.000), *post hoc* analysis indicated that the improvement in health was evident from baseline to 12 months (Wilcoxon, *p* = 0.000), but returned to its original state by 30 months. There was no significant change in pain or the incidence of pressure ulcers across the three surveys (Table 4).

**TABLE 4:** Health and employment status over time.

Employment status	Baseline	12 months	30 months	Significance over time
Percentage (number) employed	18.3% (35)	22.0% (42)*	19.4% (37)*	Cochran’s Q
				*p* = 0.000
Percentage (number) with pain on a regular basis	65.3% (125)	63.3% (121)	64.8% (145)	Friedman test NS
Percentage (number) with skin ulcers in past 12 months	15.3% (29)	8.2% (16)	12.2% (17)	Friedman test NS
Overall health (0–10 rating)	*n* = 191	*n* = 191	*n* = 140	
Median + IQ range	6.0 + 2.0	7.0 + 2.0†	7.0 + 3.0	Friedman test
Mean + SD	6.2 + 1.6	6.9 +1.8	6.2 + 2.3	*p* = 0.000

*Source*: Author’s own work

* McNemar test for change in employment, *p* = 0.000 at baseline versus 12 months and baseline versus 30 months.

† Wilcoxon Signed Rank test for change in overall health, *p* = 0.000 at baseline versus 12 months.

### Employment, income and travel

There was a small but significant increase in the reported rate of employment (Cochran’s Q, *p* = 0.000) (Table 4). The change was greatest from baseline to 12 months and was maintained through 30 months (McNemar’s, *p* = 0.000).

The increase in employment was accompanied by an increase in family income from baseline to 30 months (Table 5) (Friedman test, *p* = 0.033). The change accumulated slowly and was not significant until the 30-month mark (Wilcoxon baseline vs 30 months, *p* = 0.024). This increase applied to Peru and Uganda, but not to Vietnam.

**TABLE 5:** Family income over time.

Variable	Less than sufficient income % (*n*)	Just sufficient income % (*n*)	More than sufficient income % (*n*)
Baseline *n* = 191	47.1 (90)	45.5 (87)	7.3 (14)
12 months *n* = 191	42.4 (81)	50.8 (97)	6.8 (13)
30 months *n* = 190	36.1 (69)	57.1 (109)	6.3 (12)

*Source*: Author’s own work

Friedman test, *p* = 0.033.

Wilcoxon Signed Rank test baseline versus 30 months, *p* = 0.024.

*n*, number.

There was no reported change in overall independence. The daily distance travelled into the community changed over time (Friedman test, *p* = 0.000). Based on *post hoc* analysis, there was a pattern of increase by 12 months (Wilcoxon, *p* = 0.000) but this difference was no longer sustained by 30 months (Table 6). Of those who reported travel greater than 500 metres/day, there were 16.3% of the sample at baseline, 23.5% at 12 months and 19.4% at 30 months.

**TABLE 6:** Distance travelled over time.

*n* = 189	Less than 10 metres % (*n*)	At least 10, less than 100 metres % (*n*)	At least 100, less than 500 metres % (*n*)	At least 500, less than 1000 metres % (*n*)	At least 1000 metres % (*n*)
Baseline	24.7 (47)	36.3 (68)	22.6 (43)	2.6 (5)	13.7 (26)
12 months	14.7 (28)	23.6 (44)	38.2 (72)	14.1 (27)	9.4 (18)
30 months	17.9 (34)	38.4 (72)	24.2 (46)	6.8 (13)	12.6 (24)

*Source*: Author’s own work

Friedman test *p* = 0.000.

Wilcoxon baseline versus 12 months, *p* = 0.000.

Wilcoxon 12 versus 30 months, *p* = 0.036.

Wilcoxon baseline versus 30 months, NS.

*n*, number.

### Model of wheelchair and satisfaction

A total of 188 subjects recorded the model of wheelchair they received (Table 3) and 108 of them (57.4%) responded to the question regarding wheelchair breakdown. Reasons for incomplete data on this question are unclear and not assumed to represent a lack of breakdown. Of those using the GEN_1 model, 7.9% (6/76) reported breakdown during the previous six months compared to 6.2% (2/32) of those using the GEN_2 model.

There was no significant relationship (Pearson chi-square test for independence) between the model of the wheelchair received and the presence of pressure ulcers, pain, employment or wheelchair breakdown. There was a difference in satisfaction between GEN_1 and GEN_2 models at 12 months (Pearson chi square *p* = 0.004). Satisfaction with the GEN_1 chair averaged 7.71/10; satisfaction with the GEN_2 chair averaged 8.09. This difference in satisfaction was no longer significant after 30 months (7.65 for GEN_1 vs. 7.67 for GEN_2).

## Discussion

### Subjects

Although approximately one-third of the original sample came from each of three countries there was a difference in many of their characteristics (Table 2). Based on descriptive data, the sample from Vietnam was the eldest, had worse health and nutrition ratings, and went out of the house less frequently, appearing more debilitated. This may account for the larger than expected death rate and underscores the vulnerability of this population, particularly in less-resourced environments.

### Health status

The significant change in health at the one-year mark is consistent with results of the former study by Shore and Juillerat (2012) in Chile, Vietnam and India. They reported improved overall health after 12 months of wheelchair use, as did Toro et al. (2016) in Indonesia after 6 months of wheelchair use. However, the fact that improvement in the current study was not sustained to 30 months may be linked to factors beyond the receipt of the wheelchair (e.g. rehabilitation intervention, change in nutritional status) or linked to the pattern of skin breakdown, which, in the current study was non-significant, but appeared to move in a similar direction. These factors are worthy of future investigation.

### Employment, income and travel

The small but statistically significant change in employment is similar to that of previous reports (Grider & Wydick 2015; Shore & Juillerat 2012). In the current study, subjects described their employment as largely self-directed businesses such as selling candy, lottery tickets or food, often from their home. Employment was not always full-time; it also included selling items on weekends. Thus, the increase in employment should not be conceptualised in the usual full-time sense. There was, however, an accompanying rise in family income in Uganda and Peru, corroborating the added employment. As the baseline age in Vietnam was older (over 65 years), it is not surprising that they did not experience the same increase. While increased age does not preclude employment, the likelihood of having the necessary level of health and stamina diminishes over time.

In the baseline study sample, 47.1% of subjects reported that income was not sufficient for daily necessities (Table 5). This number declined to 36.1% by 30 months, while the number reporting sufficient income increased. This represents an important change in income earning capacity. The fact that this number remained at 36.1%, however, underlines the high risk of poverty in this population.

The rise in income was not significant at 1 year and would not have been visualised had the study ended at 12 months as in previous studies. Perhaps because the reported employment includes part-time and small self-employed enterprise, the change in income accumulated slowly, becoming significantly reported only at 30 months. Whether because of the added mobility of a wheelchair or the added stimulus of personal recognition and intervention, the potential for change in lifestyle is important.

In terms of mobility, the pattern of significant improvement at 12 months but diminishing by 30 months follows the same trend as health and incidence of pressure ulcers. The reasons for this are unclear, but warrant further study.

Compared to the mobility characteristics of residential manual wheelchair users in the United States (Tolerico et al. 2007), the changes in the current study are small. Their subjects typically travelled 2457 metres per day during an average 8.3 h of use. The difference may be because of better roads and more accessible buildings in the United States, societal norms about function or protection of those with disability or characteristics of the wheelchairs used.

### Model of wheelchair and satisfaction

Although there has been discussion in the literature over assumed health risks associated with long-term use of non-adjustable wheelchairs, this study found no sustained difference in the incidence of reported pressure ulcers or pain between the two models. It did not measure changes in posture or wheelchair manoeuvrability, which may impact long-term health and function. Because the two wheelchair models were not distributed at baseline based on levels of functional independence or overall health status, it is not possible to fully assess the effect of the wheelchair model on these variables.

It is unclear why higher satisfaction with the GEN_2 chair was expressed at 12 months while there was no difference in satisfaction between the two models at 30 months. Chair maintenance and health characteristics were not statistically different by model. There were equal subject numbers at both times, and the percentage using each model was similar (Table 3). Further exploration of this result is warranted in future studies.

Although Marchiori et al. (2015) reported similar satisfaction between users of two models of manual wheelchairs subjects commented that weight and durability were important characteristics. There was a trend towards higher satisfaction reported by Jefferds et al. (2010) in 13 recipients in India who used a prescribed wheelchair, which was lighter compared to a 50-pound depot-style chair. In the current study, the GEN_2 chair was lighter, which may account for better satisfaction at 12 months; it is unclear why this was not sustained at 30 months.

Fitzgerald et al. (2005) found that user satisfaction was linked to the number of wheelchair repairs. In a convenience sample of 130 participants, 26% reported a wheelchair repair in the previous 6 months and 27% had tyre repairs. Wheelchairs had an average age of 3.1 years, similar to the current study. Although mileage was not tracked, subjects used their chairs an average of 10.9 + 5.0 h per day. Subjects in the current study reported low rates of wheelchair repair, despite assumed poor road and transportation conditions. However, the distance travelled is much lower than in Fitzgerald’s study, perhaps accounting for the difference in repair rates. The lower distance in the current study may be because of road and sidewalk conditions being worse than in the United States, cultural norms about disability or a function of other wheelchair characteristics. As reported repair rates are low, the wheelchair models in the current study appear sufficiently robust for activity levels used by the population sample in this environment. Overall satisfaction remained favourable.

### Limitations

Because wheelchair recipients were interviewed for self-reported data, the possibility that answers were given to impress or gain approval from survey contractors cannot be excluded. No formal reliability testing of the survey has been carried out, and the study did not use controls for comparison, limiting generalisability. However, results for the final study sample were largely consistent across three countries and continents, adding validity to the results.

The subjects in Peru comprised more than 50% of the study sample. They tended to be from metropolitan areas and 75% of them received GEN_1 wheelchairs. It is not known how this may have influenced the data.

Cultural biases in the reporting of data cannot be ruled out. Varying perceptions exist about the causes of disability, and the acknowledgment of pain, health and income. Future studies should explore this contribution to the data.

## Conclusion

In three different countries on three different continents of the world, receipt of a wheelchair was associated with increased employment and income after 30 months of wheelchair use. Health status and daily distance travelled fluctuated over time. Satisfaction with the two chair models was generally favourable. Despite the more positive ratings of the GEN_2 chair at 12 months, there was no associated difference in any of the variables studied, and the higher ratings were no longer apparent at 30 months. Further investigation should be carried out to confirm these results and explore the factors responsible for fluctuating variables. This study affirms the importance of long-term follow-up of outcomes associated with wheelchair distribution in less-resourced environments.

## APPENDIX 1

### Survey questions at baseline

#### A. Demographic data

1. Date of interview and interviewer
2. Country of residence
3. Home setting
  (rural, village, large metropolitan)
4. Age, gender, height, weight
5. Education level
6. Read/write (no, basic level, advanced)
7. Employment (yes/no)
  If yes, occupation
8. Medical diagnosis or reason for use of wheelchair
9. Annual family income
  (less than sufficient to pay for basic necessities, just sufficient, more than sufficient)
10. Previous wheelchair? (yes/no)
11. Reasons for no current wheelchair
  (lack of money, no wheelchair available, wheelchair broken)

#### B. Mobility

1. Main form of transport
  (carried, crawl, scooter, crutches, bedridden)
2. Metres travelled in typical day (sum of carried, walking, riding, etc.)
  (less than 10, 9–99, 100–499, 500–999, at least 1 km)

#### C. Health status

1. Overall health (1–10; 1 = very poor, 5 = average, 10 = very good)
2. Availability of medical care in your area
  (available and affordable, available but I can’t get there, available but not affordable, there is no medical care in my area)
3. Have you had a skin ulcer in the past 12 months? (yes/no)
  If yes, how many? Describe their location
4. Do you have pain on a regular basis? (yes/no)
  If yes, severity, location
5. How often do you have adequate nutritious meals?
  (adequate quantity and nutrients every day, adequate quantity but not nutrients every day, neither adequate quantity and nutrients every day)
6. Using numbers from 1 to 10, rate your level of overall dependence on a caregiver for mobility and daily function
  (1 = total dependence, 10 = total independence)

## APPENDIX 2

### Survey questions at 12 and 30 months

#### A. Demographic data

1. Date of interview and interviewer
2. Country of residence
3. Model of FWM wheelchair received (GEN_1, GEN_2)
4. Still have and use FWM chair? If not, why? (30 month only)
5. Employment (yes/no)
  If yes, occupation
6. Annual family income
  (less than sufficient to pay for basic necessities, just sufficient, more than sufficient)

#### B. Mobility

1. Main form of transport
  (carried, crawl, scooter, FWM wheelchair, walk, bedridden, other wheelchair)
2. Metres travelled in typical day (sum of carried, walking, riding, etc.)
  (less than 10, 9–99, 100–499, 500–999, at least 1 km)

#### C. Health status

1. Overall health
  (1–10; 1 = very poor, 5 = average, 10 = very good)
2. Have you had a skin ulcer in the past 12 months? (yes/no)
  If yes, how many? Describe their location
3. Do you have pain on a regular basis? (yes/no)
  If yes, severity, location
4. Using numbers from 1 to 10, rate your level of overall dependence on a caregiver for mobility and daily function (30 months only)
  (1–10; 1 = total dependence, 10 = total independence)

#### D. Wheelchair maintenance

1. FWM wheelchair broken down within the last six months? (yes/no)
2. What parts of the wheelchair had a problem? (not including putting air in tyres)
  Front wheels, rear wheels, brakes, metal frame, plastic chair, cushion, seat rest, back rest, foot rest, harness

#### E. Satisfaction

1. Rate your overall satisfaction with the FWM wheelchair
  (1–10; 1 = very dissatisfied, 10 = very satisfied)
